# Validation of machine vision and action sport cameras for 3D motion analysis model reconstruction

**DOI:** 10.1038/s41598-023-46937-9

**Published:** 2023-11-29

**Authors:** John David Johnson, Michael Hales, Randy Emert

**Affiliations:** 1 Department of Exercise Science & Sport Management, Kennesaw State University, Kennesaw, GA USA; 2 Department of Health Promotion & Physical Education, Kennesaw State University, Kennesaw, GA USA; 3 Department of Mechanical Engineering Technology, Kennesaw State University, Kennesaw, GA USA

**Keywords:** Engineering, Optics and photonics

## Abstract

The study investigated the feasibility of using action sport cameras for motion analysis research. Data acquired from two different marker-based motion capture systems and six different camera combinations were analyzed for motion reconstruction accuracy. Two different calibration procedures were used to determine the influence on marker position reconstruction. Static and dynamic calibration mean merit score differences between the reference and experimental camera systems were 0.4 mm and 1.3 mm, respectively. Angular displacement difference between the reference and experimental camera systems range between 0.1 and 2.0 degrees. A systematic bias (− 0.54 to 0.19 degrees) was determined between the reference and the experimental camera systems for range of motion. The mean of the multi-trial findings suggests the machine vision camera system calibrated with a dynamic procedure generated highly accurate three-dimensional reconstructed ROM data (0.5 degree) followed closely by the four action sport cameras implementing a static calibration procedure (0.5 degree). The overall findings suggest the selected machine vision and action sport camera systems produced comparable results to the reference motion analysis system. However, the combination of camera type, processing software, and calibration procedure can influence motion reconstruction accuracy.

## Introduction

The study investigated the feasibility of using action sport cameras with commercial software for motion analysis research. A general perception associated with conducting high-quality motion analysis is it requires expensive professional-grade equipment to reconstruct accurate motion measurements. In contrast, the concept of using a consumer-grade camera system for producing similar results is very appealing. The idea warranted an investigation so we combined a variety of existing components, such as cameras and data processing software, in order to create and validate such a system. Our research team identified output accuracy as the priority factor for an experimental motion analysis system while considering equipment cost and transportability as essential needs. Disassembling and moving a lab-based professional motion capture system to an outdoor or off-site location has many logistical obstacles such as the risk associated with repetitive handling fragile electronic devices, and properly packaging and protecting the large quantity of parts during transportation. In addition, the possibility of outdoor environmental elements damaging equipment and lack of a power supply present additional challenges. However, there are considerable advantages associated with analyzing movement performance while in a familiar environment under natural conditions. The benefits of analyzing human or animal movement patterns in a real-world setting is a worthwhile reason for the research community to vigorously pursue designing a motion analysis system consisting of consumer grade products assuming the system can produce accurate reconstructed motion data.

A motion analysis system is an essential instrument for conducting human and animal movement analyses often used in biomechanics^1,2^, prosthetic design^3^, sport performance^4^, rehabilitation^5^, and clinical evaluations^6^. Currently, the preferred instrumentation for carrying out these motion capture operations is either optical (OP) or electromagnetic devices. These systems undoubtedly produce accurate two-dimensional (2D) and three-dimensional (3D) models. However, they are typically expensive and require additional operational needs which include extensive cabling, numerous hardware devices, and camera setup which makes these systems predominantly lab-based and not necessarily ideal for outdoor or off-site usage.

An alternative video-based technology categorized as action sport cameras (ASC) has demonstrated considerable promise in motion analysis^7–9^. These cameras have made tremendous advances in image resolution and frame rate over the past two decades, while cost has changed minimally enabling them for use in a much broader spectrum of sport and athletic performance evaluation^9,10^. This has been supported in recent literature contributions describing the application of ASC for 2D analysis^8–11^. Another camera type known as machine vision (MV) has also been used for motion analysis in recent years and is the recommended camera type for the Innovision Systems MaxTRAQ software. A MV camera is generally used in industrial settings where direct communication with a computer or robot controller is required. These cameras can perform onboard image processing which reduces energy usage and speeds up transfer of data. These cameras are less economical than most ASC but have ideal features for motion capture. The MaxTRAQ system was chosen because it is able to process images from a wide assortment of camera types including the experimental cameras we used. The processing system allows the option of storing video data directly onto the hard drive from a camera hardwired to the computer, or the ability to transfer stored video data from the camera’s onboard processor to the computer hard drive. The MaxTRAQ system provided the versatility we needed to carry out our investigation.

Extending these camera technologies for 3D sport analysis requires a scientific approach to quantify model reconstruction accuracy, camera configuration quality, data collection synchronization precision, and dedicated calibration protocols. In this study, two calibration methods are used to define a geometric space where two different motion capture systems combined with various camera configurations were analyzed for quality and accuracy. Six camera arrays totaling twenty-one cameras were focused on a work area where a motor-powered mechanical arm performed a simulated single joint movement. Calibrating the geometric space used for data capture is an essential element for ensuring the acquisition of accurate data. We will employ two calibration methods, static and dynamic. The first null hypothesis states marker position accuracy between the reference camera system and the experimental camera systems differs between calibration methods. Accuracy describes how close a calculated or measured value is to the actual value. Motion reconstruction accuracy could be influenced by camera technical specifications such as resolution and frame rate, so image quality plays a role in classifying a camera as either professional or consumer-grade. The second null hypothesis states that there is no relationship between 3D motion reconstruction data calculated from the experimental camera systems and the reference camera system. Experimental camera system accuracy will be based on the output measures compared to the output measures of the eight camera Vicon system. The third null hypothesis states that reconstructed 3D motion analysis accuracy differs between repetitions. We selected a multi-camera Vicon motion analysis system as the reference because similar setups are widely used in biomechanics and considered to be the ‘Gold’ standard in motion analysis^12–14^. The study’s aim is to determine the possibility of using consumer-grade optical cameras for producing accurate 3D motion analysis.

## Materials and methods

The 3D marker position data from the experimental hybrid motion capture systems were compared to marker position coordinates generated by the reference system. The experimental system was integrated with different camera combinations including infrared, monochrome with near-IR long pass filter, and optical for motion capture accuracy. Different ASC configurations were derived by varying the number of cameras or interchanging camera models in an array. Two different calibration instruments were included to compare calibration methods' influence on motion capture reconstruction accuracy.

### Instrumentation and design

**Figure 1:**
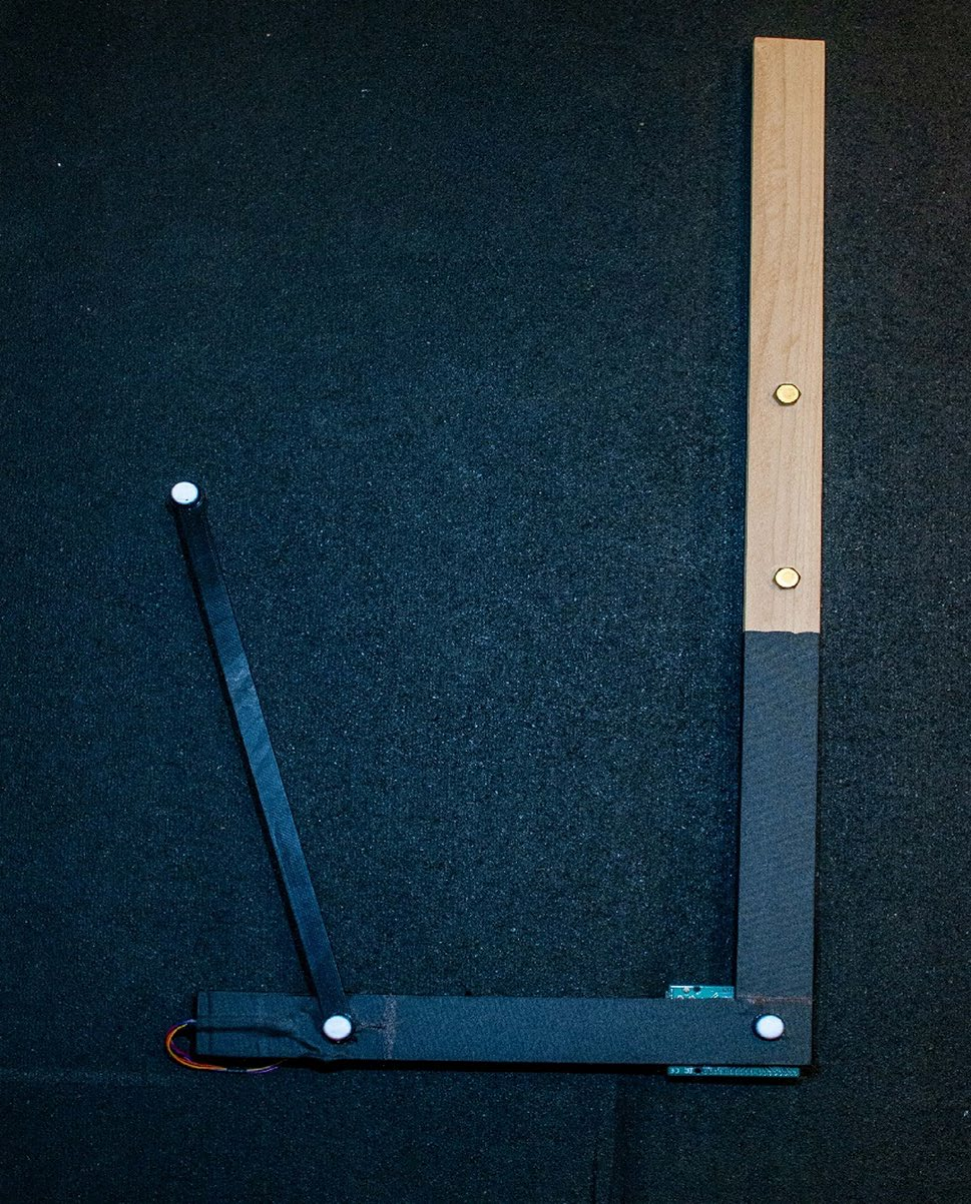
Motorized arm device.

A motorized mechanical arm was employed to enable precise motion along a singular axis. This specific motion was designed to replicate the controlled flexion and extension of a joint, operating exclusively within a defined plane (Fig. 1). The arm was comprised of two rigid segments covered with black gaffers tape to reduce light reflection from the directed LEDs illuminating the reflective markers. The mechanical arm represented a movable segment capable of producing a maximum of 90 degrees of motion (ROM). Full flexion was defined as the 90 degree position and the full extended position was defined as the 180 degree position. The arm was manually controlled by a toggle switch where the forward position produced clockwise rotation and the rearward position produced counter-clockwise rotation of the arm. The manual switching provided random range of motion between 90 and 180 to occur during each trial. The experimental methodology encompassed a series of three executed trials, each aimed at investigating the mechanical arm's motion behavior.

The mechanical arm represented a movable segment capable of producing a maximum of 90 degrees of motion. A reflective spherical marker (10 mm, 1 g) was attached to the axis (P2), and additional reflective spherical markers with the same dimensions were adhered to each segment above (P1) and below (P3) the axis. The markers used in the analysis were selected due to their practical application characteristics, such as, small diameter minimizes movement interference, light weight reduces the chance of drop-off and movement induced oscillation, and spherical shape ensures maximum surface area is in view of the cameras. The distance between the marker’s centroid for P1 and P2 was defined as segment-1 which remained stationary during the motion analysis. The distance between the marker’s centroid for P2 and P3 was defined as segment-2 which was the moving portion of the arm.

A Vicon motion analysis system was selected as the reference system for establishing baseline measurements for analyzing the experimental camera systems level of accuracy. The Vicon machine has been highly regarded in the scientific community for many years as being able to accurately reconstruct 3D movement data^12–14^. The Vicon system, in our study, incorporated eight Bonita infrared cameras integrated with Nexus 2.8 processing software (Vicon Motion Systems, Inc., USA). The Vicon Bonita cameras (V-8) were hardwired to a synchronization hub prior to transferring data to a central computer for 3D model reconstruction. A separate infrared camera configuration comprised of four Vicon Vero 2.2 motion capture cameras (V-4) served as an experimental system. These V-4 cameras were tripod mounted in the same location as the other experimental camera arrays. The V-4 cameras were hardwired to the lab-based OP motion capture system (Vicon Motion Systems, Inc., USA) and synchronized using the same procedure as the V-8 setup. The motion capture system (Innovision Systems, Inc., USA) capable of using infrared or optical cameras consisted of four Sentech USB 3.0 machine vision (MV-4) monochrome cameras with infrared lights attached to illuminate the markers (Omron Sentech Co., USA). The MV-4 cameras were hardwired to a computer for synchronization and controlled using the MaxTRAQ 3D software (Innovision Systems, Inc., USA). A fourth camera array consisted of four action sport cameras varying in model (GoPro, Inc., USA) also utilized the MaxTRAQ 3D data processing software. The quantity and model of the optical cameras selected for analysis (three GoPro Hero 5 Black, one GoPro Hero 4 Silver, and one GoPro Hero 9) could be grouped in a manner so cameras with similar technical specifications could be compared but also cameras with different technical specifications could also be grouped and compared. A mini-LED cube light was located at each ASC location for additional marker illumination. A separate LED light controlled by an Arduino was triggered to time-stamp frame-one for each ASC recording to insure proper frame synchronization. Both motion analysis software packages utilized automatic marker tracking to provide consistency of locating marker center across trials.

All cameras in the study were set to record 120 frames-per-second (fps). Each camera used with the reference system was manually focused to ensure optimal image viewing precision. The MV-4 and ASC have a fixed focal length, however, the cameras have settings which can be selected to guarantee optimal quality for a particular setting. The field of view (FOV) for each ASC was set at “Narrow” to minimize picture distortion. The shutter speed on the GoPro Hero 5 Black and Hero 9 cameras was set to 240 while the GoPro 4 Silver was set to “Auto”. To control aperture all action sport cameras were set to an ISO of 1600. The resolution for the GoPro Hero 4 and 5 was 720 p while the Hero 9 was 1080 p. The camera settings were predominantly determined through trial and error. The frame rate of 120 fps was the highest that the ASC’s could capture. This also was an acceptable frame rate for the other systems in the study. Having all cameras set at 120 fps eliminated the need to manipulate the data after collection. Due to the lighting and the small sensor size of the ASC’s shutter speed, ISO needed to be controlled to reduce motion blur. It is accepted to double the shutter speed of the camera’s frame rate in order to eliminate motion blur. An ISO 1600 was determined to allow the greatest amount of light into the ASC’s camera with acceptable noise. The settings selected for the analysis were determined to be optimal for the environment in which data were collected, however the settings may not be the best for other scenarios.

### Camera calibration protocol

The motion capture systems generated system-specific values to quantify calibration accuracy. The Vicon motion capture camera setups were calibrated using a dynamic calibration protocol recommended by the manufacturer which included a rigid T-shaped device equipped with LED lights of known distances. Vicon camera arrays V-8 and V-4 were calibrated concurrently to ensure the defined geometric volume was identical for each camera configuration. More specifically, the V-8 and V-4 camera arrays generated camera residuals which were the root mean square difference of the strobe ring center to the centroid and the reflection of the ray from the marker centroid to the camera lens^15^. According to the manufacturer, a camera residual error (< 0.25 mm) signified an accurate 2D contribution by that camera and an acceptable calibration procedure which served as the calibration qualifying threshold^15^.

**Figure 2:**
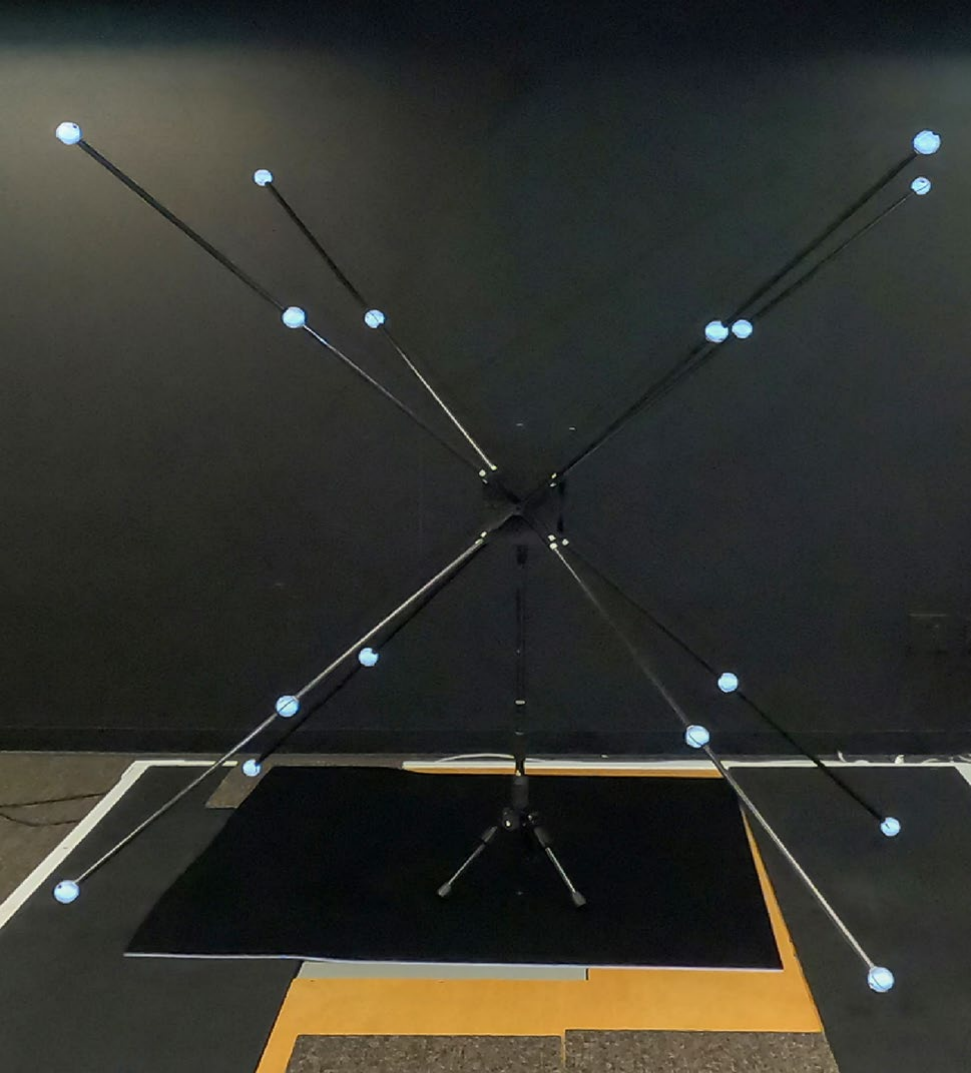
16-point calibration frame for static calibration.

The Innovision Systems MaxTRAQ 3D software provides an option to use either static or dynamic generated coordinates for calibration. Static calibration uses a three-axis apparatus, called a frame, which has reflective markers attached in predetermined locations with known three-dimensional coordinates. The custom frame was designed using CAD software (SolidWorks Corp., USA) and several components were printed with polylactic acid filament (KSU 3D Print Center, USA). The frame consists of eight carbon fiber tubes extending from a centroid cubical hub. The assembled structure measures 1.8 m in height, 1.1 m in width, and 1.8 m in length for a cubic volume of 3.6 m, is centrally supported by a monopod. Sixteen spherical reflective markers, 14 mm in diameter, are attached to the tubes with two markers per extension tube spaced 0.67 m apart (Fig. 2). In order to calibrate a space while simultaneously using multiple camera combinations our unique camera mount system allowed us the opportunity to create three different ASC combinations for analysis. We classified these as ASC-1, ASC-2, and ASC-3. Camera combination ASC-1 consisted of three GoPro Hero 5 Black and one GoPro Hero 4 Silver; ASC-2 included three GoPro Hero 5 Black and one GoPro Hero 9; and ASC-3 consisted of three GoPro Hero 5 Black only. For static calibration, the custom frame was used with a direct linear transformation calculation internal to the MaxTrac 3D software. Summary of the DLT calibration for achieving the merit value (Supplementary Fig. S1).

For dynamic calibration, we used the manufacturer’s supplied calibration fixture, called a wand, which consists of a rigid T-shaped handheld device with a 14 mm spherical reflective marker attached on each end of the main crossbar with a known distance between markers. In addition, a stationary rigid L-frame with four 14 mm reflective markers attached at known locations along the x and y axis was placed on the floor in the workspace to define x, y, z orientation. The approximate dimensions of the calibrated volume was 1.8 m in height × 1.1 m width × 1.8 m in depth. The MaxTRAQ program for dynamic calibration followed a similar procedure as the Vicon motion analysis system, however, one difference was marker position accuracy which is reported as a merit score. The merit score is the root-mean-square (RMS) of all individual marker merits expressed in millimeters (mm). A merit score < 1 mm is recommended for the dynamic calibration procedure in a 1 m volume. A merit score < 10 mm signifies an acceptable static calibration procedure, however, a target value closer to one is preferred. As workspace volume increases, merit score allowance increases. The geometric space used in our study for data collection was approximately 3.6 cubic meters.

### Data acquisition procedure

**Figure 3:**
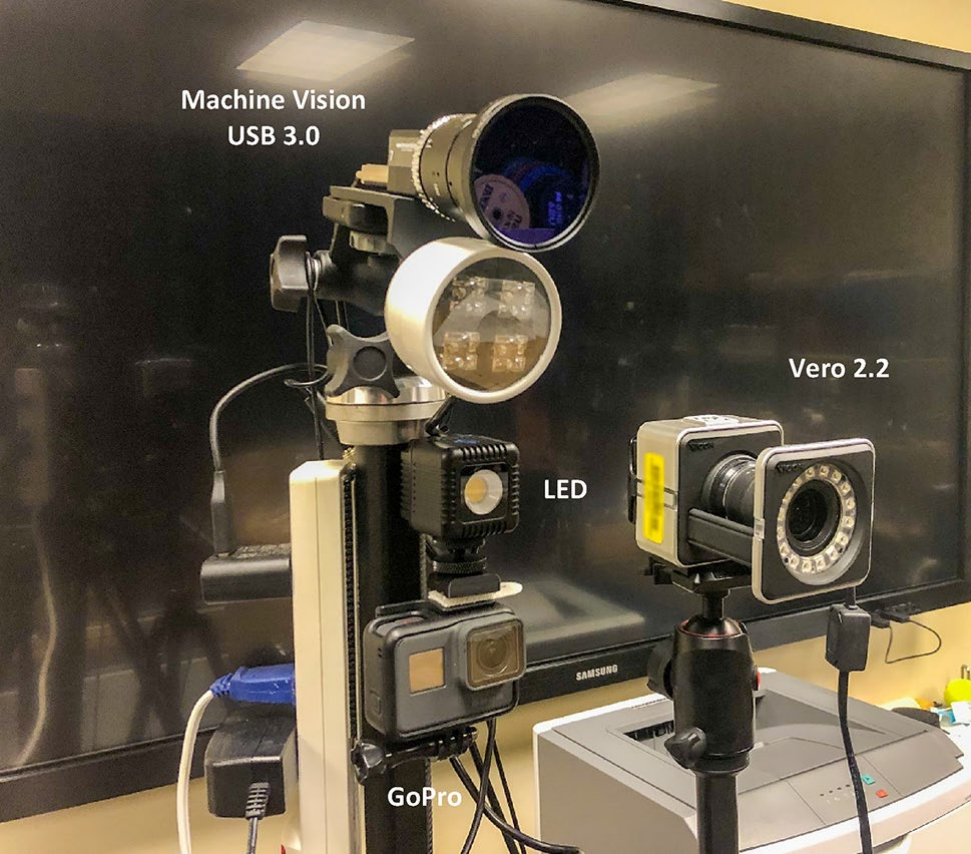
The experimental camera setup.

The V-8 cameras were wall mounted, eight feet above the floor, around the perimeter of the calibrated space. Four tripod mounted camera stations were arranged in a semicircular manner approximately 50 degrees apart. Each camera station consisted of a Vicon Vero 2.2 camera, a GoPro Hero (4, 5 and/or 9) camera, a Sentech USB 3.0 camera with an infrared light, and a mini-LED light fixture (Fig. 3). Prior to data collection, the reflective-marker wand was processed to verify that an acceptable quality calibration was performed. The V-8, V-4, and MV-4 were hardwired synchronized and triggered by a manual keystroke. The ASC’s were triggered using an app that started all of the cameras recording. A light, in view of the ASCs, was then turned on and off to mark a frame which can be used to align the ASC video during data processing. The motorized arm produced a simulated joint flexion–extension cycle. Marker coordinates of the V-8 and V-4 motion data were processed using Vicon Nexus 2.8, the MV-4, ASC-1, ASC-2 and ASC-3 video recordings were processed in MaxTRAQ 3D. The arm motion recordings from the ASC and MV-4 camera arrays were processed twice, once after the static (frame) calibration protocol and once after the dynamic (wand) calibration was applied. The mechanical arm motion analysis was conducted using a variety of camera and calibration combinations: V-8 (wand), V-4 (wand), MV-4 (wand), MV-4 (frame), ASC-1 (wand), ASC-1 (frame), ASC-2 (wand), ASC-2 (frame), ASC-3 (wand), and ASC-3 (frame). All twenty-one cameras recorded the arm’s movement simultaneously. Data were extracted using Nexus 2.8 for the Vicon cameras and MaxMATE motion analysis toolbox for further MV-4 and ASC evaluation (Innovision Systems, Inc. USA).

### Statistical analysis

Descriptive statistics were used to analyze 3D model reconstruction accuracy. Statistical error was used to quantify accuracy, which we defined as the difference between the measured value and the actual value. Means and standard deviations were calculated to analyze the dependent variables: merit score (mm), angular displacement (degree) and segmental length (mm). The independent variables were camera system type (V-8, V-4, MV-4, ASC-1, ASC-2, and ASC-3), calibration method (static and dynamic), and trial (T1, T2, T3). The Shapiro–Wilk test was used to test distribution normality. The comparison of 3D reconstructed motion output data from the reference system and experimental camera systems were used to calculate a root-mean-square error (RMSE) value for each combination of camera system and calibration method. For validating the experimental camera systems, a Pearson’s correlation coefficient (r) was used to analyze the relationship between the reference camera system output data set and the experimental camera systems output data set when incorporating both calibration methods. For camera reliability across trials, a three-way ANOVA was used to describe group mean comparisons between experimental camera systems, calibration type, and trials relative to the reference system (V-8). The concurrent capture of arm motion was also used to present reliability (intra-class correlation coefficient – ICC) between experimental camera systems and the reference system for three trials. We set alpha at 0.01 since we were investigating accuracy between instruments and tight margins were very important to us. A Bland–Altman technique was used to analyze agreement between the individual camera systems V-4 (wand), MV-4 (frame), MV-4 (wand), ASC-1 (frame), ASC-1 (wand), ASC-2 (frame), ASC-2 (wand), ASC-3 (frame, ASC-3 (wand) relative to the reference system (V-8).

## Results

The Shapiro–Wilk test rejected the null hypothesis for normality. Consequently, the Box-Cox technique was applied to the non-normal data prior to statistical analysis. Table 1 reports static and dynamic merit scores for each experimental camera system. The reference system calibration residual errors for Bonita cameras 1 through 8 were: 0.11 mm, 0.09 mm, 0.09 mm, 0.08 mm, 0.10 mm, 0.08 mm, 0.10 mm, and 0.11 mm, respectively. The V-8 camera system RMS (merit score) was 0.09 mm which is below the calibration qualifying threshold.

**Table 1:** Static and dynamic calibration merit scores of accuracy for the experimental camera systems.

Camera system	V_1_	V_2_	V_3_	V_4_	G5_1_	G5_2_	G5_3_	G4	G9	MV_1_	MV_2_	MV_3_	MV_4_
Static calibration merit score (mm)													
V-4	–	–	–	–	–	–	–	–	–	–	–	–	–
ASC-1	–	–	–	–	1.57	1.68	1.42	1.29	–	–	–	–	–
ASC-2	–	–	–	–	1.57	1.68	1.42	–	1.59	–	–	–	–
ASC-3	–	–	–	–	1.57	1.68	1.42	–	–	–	–	–	–
MV-4	–	–	–	–	–	–	–	–	–	1.10	1.26	1.29	1.21
Dynamic calibration merit score (mm)													
V-4	0.08	0.20	0.24	0.09	–	–	–	–	–	–	–	–	–
ASC-1	–	–	–	–	1.75	2.31	1.11	1.23	–	–	–	–	–
ASC-2	–	–	–	–	2.01	1.82	2.23	–	1.86	–	–	–	
ASC-3	–	–	–	–	1.94	2.04	1.31	–	–	–	–	–	–
MV-4	–	–	–	–	–	–	–	–	–	0.49	0.48	0.49	0.45

V-4 used dynamic calibration only. Merit score represents the RMS. Merit score < 10 mm signified an acceptable static calibration procedure. Vicon merit score < 0.25 mm and MaxTRAQ merit score < 1 mm signified an acceptable dynamic calibration procedure. V-8 merit score = 0.09 mm. The closer the merit score is to 0, the more accurate the camera reconstruction measurement is to the actual measurement.

A repeated measures two-way ANOVA calculation between calibration method and camera system type (F = 1.01, p = 0.391) failed to show an interaction between independent variables. Descriptive measures for camera system and calibration method were calculated to quantify camera system reconstruction output quality based on merit score means and standard deviations (Supplementary Table S1).

### 3D model reconstruction analysis

Table 2 reports RMSE scores which describe camera system output accuracy, with static (S) and dynamic D) calibration, for each experimental camera system compared to the V-8 system output data. Further analysis investigated a linear relationship between the reference system and the experimental camera systems for the three trials. A single arm movement expended 3.3 s yielding 398 data points for analysis. The dependent variable was angular displacement and the experimental camera systems were analyzed when incorporating both calibration methods. The Pearson’s r calculations for Trial 1 r(398) = 0.98, p < 0.001); Trial 2 r(398) = 0.98, p < 0.001); and Trial 3 r(398) = 0.99, p < 0.001) suggest a strong relationship exists between the experimental camera systems and the reference system. Intra-class correlation calculation for camera systems across trials was > 0.9.

**Table 2:** Reconstructed angular displacement differences between the experimental camera system and the reference camera system across trials.

Camera system	Calibration method	RMSE (degree)			RMSE (degree), mean (SD)
		Trial 1	Trial 2	Trial 3	
V-4	Dynamic	0.49	0.22	0.34	0.35 (0.13)
ASC-1	Static	0.29	0.53	0.57	0.46 (0.14)
ASC-1	Dynamic	0.42	0.57	0.61	0.53 (0.09)
ASC-2	Static	0.55	0.54	0.60	0.57 (0.03)
ASC-2	Dynamic	0.60	0.57	0.63	0.60 (0.03)
ASC-3	Static	0.47	0.76	0.57	0.60 (0.11)
ASC-3	Dynamic	0.69	0.57	0.57	0.61 (0.14)
MV-4	Static	0.66	0.38	0.43	0.49 (0.15)
MV-4	Dynamic	0.68	0.42	0.47	0.52 (0.01)
ROM mean	–	77.1	72.4	82.1	–

Root-mean-square error (RMSE). Range of motion (ROM). ASC-1 (3 GoPro 5 s + 1 GoPro 4), ASC-2 (3 GoPro 5 s + 1 GoPro 9), ASC-3 (3 GoPro 5 s), MV-4 (4 Sentech USB 3.0). Wand = dynamic calibration and Frame = static calibration.

Segment-1 length was 145.5 mm, where segment-2 length was 288.0 mm. The stationary segmental lengths reconstructed by the Vicon-8 system were 145.5 mm and 288.0 mm, respectively. Table 3 reports predicted segmental length differences presented as a percentage error between the reference system and experimental camera systems. Both static (S) and dynamic (D) calibration calculations were incorporated to analyze motion output accuracy (Supplementary Table S2).

**Table 3:** Segmental length differences between the experimental camera systems and the reference system while implementing both calibration procedures across three trials.

	Reference (mm)	Percentage error (%) using dynamic calibration					Percentage error (%) using static calibration			
	V-8	V-4	ASC-1	ASC-2	ASC-3	MV-4	ASC-1	ASC-2	ASC-3	MV-4
Segment 1										
T1	145.60	− 0.12	− 0.69	− 0.81	− 0.68	− 0.12	1.19	0.88	1.22	0.43
T2	145.56	− 0.09	− 0.63	− 0.96	− 0.69	− 0.18	1.17	0.72	1.15	0.39
T3	145.57	− 0.13	− 0.60	− 0.76	− 0.54	− 0.28	1.25	0.91	1.29	0.34
Segment 2										
T1	287.86	0.22	− 0.35	− 0.60	− 0.89	− 0.54	1.88	1.57	0.97	1.85
T2	287.75	0.27	− 0.34	− 0.48	− 0.81	− 0.47	1.86	1.63	1.03	1.86
T3	287.95	0.22	− 0.47	− 0.72	− 1.00	− 0.59	1.80	1.47	0.92	1.74

Segment-1 = distance between points 1 and 2, Segment-2 = distance between points 2 and 3.

**Figure 4:**
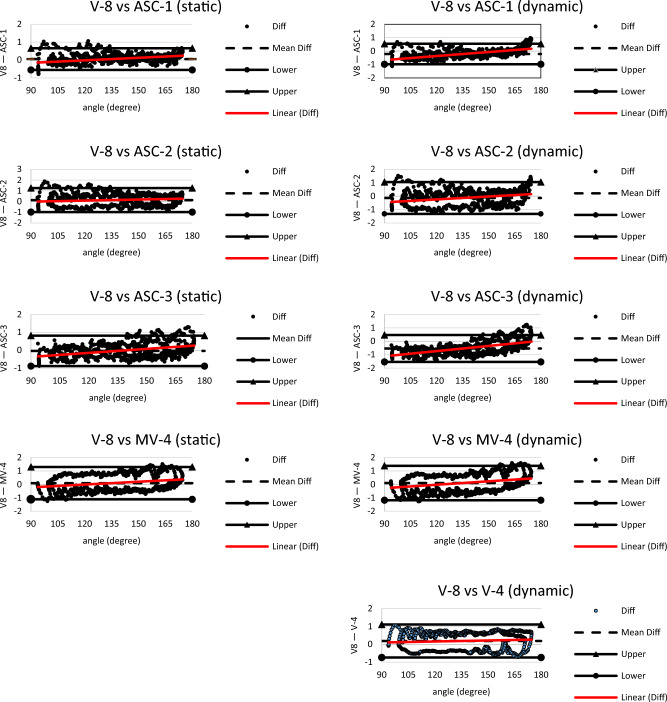
Angular displacement bias between the reference system and the experimental systems. ASC-1 (3 GoPro 5 s + 1 GoPro 4), ASC-2 (3 GoPro 5 s + 1 GoPro 9), ASC-3 (3 GoPro 5 s), MV-4 (Sentech USB 3.0). static (frame calibration) and dynamic (wand calibration).

Figure 4 shows the Bland–Altman assessment of correspondence plots to the experimental camera setups relative to the reference motion capture system (V-8). The absolute systematic bias ranged from 0.03 to 0.54 degrees. The Bland–Altman bias for the systems incorporating a dynamic calibration method (*M* = − 0.12, SD = 0.29) and the systems incorporating a static calibration method (*M* = 0.003, SD = 0.16). The upper (*M* = 0.87, SD = 0.44) and lower (*M* = − 1.15, SD = 0.31) limits for the camera systems incorporating a dynamic calibration method were compared to the upper (M = 1.00, SD = 0.32) and lower (M =  − 0.91, SD = 0.25) limits for the camera systems incorporating a static calibration method.

Three arm motion trials using angular displacement were used to measure repeatability of the motion output data (Table 4). We performed each analysis three times to test reliability of the experimental camera systems. A repeated three-way ANOVA calculation determined no interaction (F = 0.22, *p* = 0.802) was found for calibration method and camera system type analyzing motion for a series of consecutive trials. Intra-class correlation for angular position reliability incorporating both calibration methods was 0.92 with 95% confidence interval = 0.7–0.8. Further analysis investigating angular position data determined ICC = 0.94 with 95% confidence interval = 0.7–0.8 for V-4, MV-4, ASC-1, ASC-2, and ASC-3 across trials.

**Table 4:** Descriptive measures for ROM across experimental camera systems relative to the V-8 system.

Camera system	V-8	V-4	ASC-1	ASC-1	ASC-2	ASC-2	ASC-3	ASC-3	MV-4	MV-4
Trial 1										
Calibration method	Wand	Wand	Frame	Wand	Frame	Wand	Frame	Wand	Frame	Wand
Min angle (degree)	94.08	93.97	94.25	94.53	94.31	94.61	94.37	94.88	94.48	94.31
Max angle (degree)	171.26	170.97	171.03	170.91	171.09	76.38	170.91	171.03	170.86	170.62
ROM (degree)	77.12	77.01	76.77	76.32	76.78	76.57	76.55	76.09	76.38	76.32
Error diff (degree)	–	0.137	0.653	0.785	0.928	0.756	0.785	1.02	0.733	0.734
Trial 2										
Calibration method	Wand	Wand	Frame	Wand	Frame	Wand	Frame	Wand	Frame	Wand
Min angle (degree)	100.49	99.01	99.41	98.94	99.29	100.84	100.81	101.29	100.78	100.84
Max angle (degree)	172.92	171.89	172.52	172.46	172.17	172.35	172.57	173.21	172.06	172.52
ROM (degree)	72.42	72.88	73.11	73.45	72.88	71.45	71.73	71.85	71.28	71.68
Error diff (degree)	–	− 0.0181	− 0.475	− 1.04	− 0.475	0.939	0.664	0.578	1.02	1.26
Trial 3										
Calibration method	Wand	Wand	Frame	Wand	Frame	Wand	Frame	Wand	Frame	Wand
Min angle (degree)	93.16	92.93	93.51	93.97	93.33	93.73	93.79	94.37	92.93	92.82
Max angle (degree)	175.38	174.87	174.52	174.01	174.47	173.84	174.58	174.59	174.92	174.69
ROM (degree)	82.16	81.81	81.02	79.98	81.13	80.09	80.79	80.16	81.99	81.87
Error diff (degree)	–	0.338	0.825	0.985	− 1.09	1.02	1.07	0.618	− 0.876	0.097

Root-mean-square error (RMSE). Range of motion (ROM) measured in degrees. Angle and relative difference values are reported in degrees. ASC-1 (3 GoPro 5 s + 1 GoPro 4), ASC-2 (3 GoPro 5 s + 1 GoPro 9), ASC-3 (3 GoPro 5 s), MV-4 (4 Sentech USB 3.0). Wand = dynamic calibration and Frame = static calibration.

## Discussion and implication

Our study addressed this issue regarding output data accuracy when using machine vision and action sports cameras for 3D motion analysis. The notion of using these cameras for analyzing motion has been investigated in the past providing supportive or inconclusive findings for their use. One study investigating the use of action sport cameras for motion analysis reported 3D model reconstruction accuracy to be less than 3 mm, nonetheless a significant difference was observed between the reference system (Vicon) and the experimental motion analysis system^7^. A similar study incorporating action sport cameras with a custom calibration procedure reduced the average 3D reconstruction error to 2.5 mm (1280 × 720 p) and 1.5 mm (1920 × 1080 p)^8^. Another study found that the average error of the reconstructed marker distances was less than 1.5 mm on average across the data collection geometric volume^8^. A study investigating repeatability of action sport cameras found a very low variability 1.7% and 2.9% for in-air and underwater analyses, respectively^11^. Lastly, a study comparing an unspecified motion capture system to a Vicon motion analysis system found a significant difference between system kinematic calculations. Marker placement abnormalities, different calibration procedures, and data processing differences could have impacted the outcome measures of the study^10^. The conflicting outcomes do suggest further investigation was needed. Regardless, the limitations presented in each of these studies were taken into consideration during the designing phase of our methodological procedures. We believe applying the recommendations put forth from previous studies of similar magnitude contributed to our encouraging outcomes. A key element of our study centered on comparing a group of budget-friendly consumer-grade camera configurations to a traditional high-quality motion capture system. A Vicon motion analysis system integrated with eight Vicon cameras generated the reference output measurements. The MaxTRAQ 3D software was selected as the experimental motion capture system because of, its ability to produce accurate data, versatility to use a diverse array of cameras and calibration methods while also meeting our budget criteria.

### Camera calibration comparison

Proper calibration is extremely important for ensuring accurate 3D model reconstruction. The calibration comparison analysis included a static and dynamic method which incorporated a fixed-marker custom frame or t-shaped wand both with known reflective marker coordinates. The first step of the analysis was to determine that an acceptable calibration was achieved for each of the camera systems. The reference system (V-8) mean residual error was 0.09 mm which signified a high-quality calibration procedure was achieved. The experimental camera systems were calibrated using both calibration procedures and each system achieved an acceptable outcome. An aspect of the study sought to determine the influence a calibration method has on 3D reconstruction accuracy when using consumer grade cameras for data capture. To properly analyze the operation, we compared 3D model reconstruction following the dynamic and static calibration procedures for each camera array. For static calibration, a merit score for the experimental camera systems ranged between 1.2 and 1.6 mm. For dynamic calibration, the experimental camera merit score ranged between 0.5 and 1.8 mm. The mean RMS for the V-4 camera system was 0.15 mm comparative to the V-8 camera system. ASC-1 produced a slightly more accurate calibration than ASC-2 or ASC-3, possibly due to the GoPro 9 camera’s higher resolution factor. The motion analysis indicated ASCs generated more accurate outcome measures when coupled with static calibration, conversely, the MV-4 cameras provided more accurate data coupled with the dynamic procedure. This led us to believe the differences could be due to the machine vision camera’s ability to process image data internally.

The first null hypothesis states marker position accuracy between the reference camera system and the experimental camera systems differ between calibration methods. The error difference between camera system types and the reference system was not statistically different, thus rejecting the first null hypothesis. The unexpected statistical outcome could be attributed to the small residual error demonstrated by the reference camera systems, combined with the small error variance demonstrated between experimental camera systems. Regardless, a mean merit score difference of 0.4 mm for static calibration and 1.3 mm for dynamic calibration indicates a respectable level of accuracy for the consumer grade cameras compared to the professional motion analysis system. While not significant, the dynamic (wand) procedure was marginally favored by the V-4 and MV-4 cameras, as a result, producing more accurate reconstructed 3D position data. In contrast, the ASC systems produced less accurate dynamic calibration data. This could be associated with the number of frames processed for each method. Static calibration utilized one image from each camera for reconstructing marker location Dynamic calibration required fifteen seconds of wand motion which resulted in the processing of 1800 frames for each camera view. The difference in frame processing between static and dynamic procedures could potentially increase error probability in locating marker centers, however, it should be noted the difference was not statistically significant. It should also be noted that the reprocessing of marker locations on the frame across camera systems was more consistent than locating marker locations during dynamic calibration process. Most importantly, both calibration methods yielded small merit scores suggesting both procedures could be used with confidence for producing accurate 3D motion calculations. There are advantages and disadvantages associated with each method. The dynamic method provides convenience of use and easily allows defining a larger volume for data collection, however, processing the video for each camera could be time consuming. Depending on the number of cameras used and data processing software, this process could be extremely time consuming. The static calibration method takes time to set up and the data capture volume is limited to the size of the frame. The calibration step is completed quickly since a single image from each camera is all that is required for processing.

### 3D model reconstruction accuracy

The second null hypothesis states that there is no relationship between the 3D motion output data calculated from the experimental camera systems and the reference camera system. The statistical analysis rejected the second null hypothesis and there are several findings worth discussing regarding accuracy of the experimental camera systems. While not significant, the V-4 system produced the most accurate reconstructed values of all experimental camera systems compared to the reference system (< 0.4 degree). This was not surprising since both systems were considered high quality professional-grade. Based on the small difference between camera configurations, one could argue that eight professional-grade cameras provided slightly more accurate results compared to four professional-grade cameras. The consumer-grade camera system demonstrating calibration quality and angular motion accuracy closest to the V-8 system was the MV-4 camera setup employing the dynamic calibration protocol (< 0.5 degree). The most accurate action sport camera configuration for determining joint movement was ASC-1 implementing the frame calibration (< 0.5 degree). Interestingly, each of the ASC combinations demonstrated a more accurate 3D movement reconstruction when using the 16-point frame for calibration. Action sport camera technology could be partially responsible for the phenomenon. The ASC records 2D visual images while reflective markers are illuminated by an external light source. During arm movement, light reflection on the markers could impact the accuracy of processing the marker centroid thus affecting the quality of the 3D constructed models. Marker light reflection also could account for some accuracy differences observed between the frame and wand residual errors. Another notable finding indicated the experimental camera systems incorporating the dynamic calibration method tended to underestimate the reconstruction data compared to the reference system. In contrast, the experimental camera systems incorporating the static calibration method showed a trend to overestimate the reconstruction data compared to the reference output data. Pearson’s correlation analysis provided additional evidence of a strong relationship (r > 0.97) between the experimental camera systems and the reference system (V-8) when measuring angular displacement.

A systematic bias for angular displacement was determined between the reference and experimental systems ranging between − 0.54 and 0.19 degrees which is considered a relatively small error. It is understandable that some bias would be present since different camera technology exists between the reference camera system and the experimental cameras. The Vicon system utilizes standard Infrared technology, whereas ASC uses LED lights and MV-4 monochrome utilizes near-IR technology for illuminating the reflective markers. Another potential factor contributing to the error between systems was the fact that the Vicon cameras produce a higher resolution (1920 × 1080) than the GoPro cameras and USB3 cameras. The Bland–Altman plots also indicate slight differences existing between the different experimental camera systems. These differences could be attributed to different light sources and individual camera intrinsic such as resolution, aperture, or the cameras field of view. For instance, V-4 included four Vero cameras with 1280 × 1024 resolution, ASC-1 camera setup consisted of three GoPro Hero 5 Black cameras and one GoPro Hero 4 Silver camera each with 1280 × 720 p resolution, ASC-2 consisted of three GoPro Hero 5 Black cameras with 1280 × 720 p resolution and one Go Pro Hero 9 camera with 1920 × 1080 p resolution, ASC-3 consisted of only three GoPro Hero 5 Black cameras with 1280 × 720 p resolution, and the MV-4 camera setup consisted of four Sentech USB 3.0 cameras with 1600 × 1200 p resolution. Each experimental camera field of view was set to narrow and capture rate was set to 120 fps. Even though no significant difference was found, the four GoPro cameras (ASC-1) with the same resolution settings (720 p) produced slightly more accurate mean merit scores than three GoPro cameras with the same resolution settings (720 p). Combining GoPro cameras in a system with different resolutions (ASC-2) produced less accurate 3D model reconstruction than the configurations consisting of the same resolution settings (ASC-1 and ASC-3), however, the differences were not statistically significant. Aside from the V4- setup, the most accurate experimental camera configuration was produced by the higher resolution USB 3.0 camera (MV-4) setup.

### 3D model reconstruction repeatability

The third null hypothesis states that reconstructed 3D motion analysis accuracy differs between repetitions. Relative ROM difference between the experimental camera system and the V-8 system was used to summarize accuracy across trials. The statistical analysis rejected the third null hypothesis suggesting no significant difference was observed for ROM in 3D reconstruction accuracy between trials 1–3. This indicates, for the small trial set, the systems measured with a relatively high degree of accuracy across trials. Further analysis highlighted two important findings. First, angular position reliability was very high (ICC > 0.90) for both static (frame) and dynamic (wand) methods. Second, angular position demonstrated very high reliability (ICC > 0.90) for V-4, MV-4, ASC-1, ASC-2, and ASC-3 between trials 1–3. Thus, we conclude that an ICC > 0.90 indicates that the reference coordinates can be rated with “excellent” reliability by the different experimental camera setups.

In conclusion, the aim of the study was to determine the likelihood of using consumer grade cameras to generate accurate video for 3D motion analysis. Overall, our study successfully demonstrated machine vision and action sport cameras can produce accurate 3D reconstructed measurements while achieving our secondary goals of low cost, portability, mobility, and ease of set up. Besides the V-4 setup, all other experimental camera configurations utilized the MaxTRAQ 3D software and demonstrated a high degree of 3D model reconstruction accuracy. The authors acknowledge the existence of other reputable image processing motion capture systems along with several open-source solutions. While literature on motion reconstruction analyses incorporating the MaxTRAQ 3D system is limited, the Innovision system met several key criteria for the research study (i.e. commercial availability, versatile and accurate). The motion analysis system was chosen because it is able to process images from a wide assortment of camera types including the experimental cameras used in the current study. In addition, the system allowed the option of storing video data directly onto the hard drive from a camera hardwired to the computer, or the ability to transfer stored video data from the camera’s onboard processor to the computer hard drive. The feature was a necessity for incorporating the selected ASCs. In addition, MaxTRAQ 3D accepts both static and dynamic calibration protocols which was an important aspect of the research study. Overall, the MaxTRAQ 3D system provided the versatility we required to carry out our investigation. A noteworthy finding from the study suggests a properly paired MV or ASC system, processing software, and calibration procedure can produce a high degree of reconstruction accuracy where less than 1% error difference separated the measurements from the reference system. For example, the absolute average of the multi-trial results suggest the MV-4 camera system calibrated with the wand generated highly accurate 3D reconstructed ROM data followed closely by the four GoPro cameras (ASC-1) employing the frame calibration. However, one noted difference between the MV-4 and ASC systems is connectivity. The MV-4 setup requires cameras and computer to be hardwired for synchronization and camera control. The action sport cameras store recorded video onboard without connecting to a motion capture system, so it is not necessary to have an on-site computer or cabling. For power needs, we mounted portable battery storage units on the tripods to power the camera and mini lights. Portability and mobility were easily accomplished since the MV-4 and ASC systems consisted of only four light-weight tripods, four small cameras, a 16-point calibration frame that disassembles easily, four small portable power banks, and one hand-held computer for synchronizing and camera control. Another difference between the MV-4 and ASC configurations is camera cost. The MV-4 camera system costs less than a traditional motion capture system but more costly than action sport cameras. We also elected to 3D print and build our calibration frame using commercial parts for cost effectiveness and ease of manufacturing accessibility. For the final phase of the analysis, we decided to conduct a simple qualitative assessment to address the characteristics of an ASC system. We were able to set up and calibrate the experimental system in less than twenty minutes. Considering the portability, ease of setup, efficiency of data capture, and relative low cost of a motion capture system consisting of action sport cameras should be considered as an option for biomechanical analyses. Further investigation in using the MV-4 and ASC systems for outdoor analyses is needed to quantify 3D model reconstruction accuracy.

### Limitation of the study

Consumer based camera usage for motion analysis presented several limitations. Due to the lack of real time calibration quality feedback from the MaxTRAQ 3D software, we chose to record and process the calibration recordings on location at the beginning of the data collection session to ensure a high-quality calibration was conducted. This process was time-consuming but necessary because discovering poor calibration after data collection was completed would disqualify the motion data acquisition for that session. Simply considering the actual outputs, the action sport cameras were not as accurate as the high-end motion capture cameras used in the study. Also, within the statistical significance confines (alpha = 0.01), the ASC systems produced acceptable outcomes relative to the V-8 and V-4 systems. However, to achieve these highly correlated results we learned the importance of properly illuminating the reflective markers. This potentially could be an issue with optical cameras used outdoors where natural light is dependent on many uncontrollable variables. One last limitation was the use of only a singular plane movement. Highlighting potential limitations of our methodology provides important guidance for future work to gain insight into the feasibility of using MV-4 and ASC for research purposes.

## Future works

In the study, we incorporated 6 different camera arrays, two different calibration procedures, two different motion analysis systems and a motor-driven testing apparatus. The objective of the investigation was to analyze various consumer camera configurations and setups to identify the most accurate combinations for motion analysis. Overall, the preliminary findings were promising, and our next step in the process is to design an experiment with human participants to explore MV-4 and ASC validity in the context of whole-body 3D kinematic analysis in an outdoor setting. The extenuation of the present study will include multi-axis movements such as walking, running, lifting, and throwing activities in real-world settings.

### Supplementary Information

Supplementary Figure S1.

Supplementary Table S1.

Supplementary Table S2.

## Data Availability

The datasets generated during and/or analyzed during the current study are available from the corresponding author on reasonable request.
