# Supplementary material for: Validation of machine vision and action sport cameras for 3D motion analysis model reconstruction
Source: Sci Rep. 2023 Nov 29;13:21015. doi: 10.1038/s41598-023-46937-9 (PMC10687061; doi:10.1038/s41598-023-46937-9)
Supplement: Supplementary file 1 — Supplementary Figure S1. [file 41598_2023_46937_MOESM1_ESM.docx]

**FIGURE S1:** Direct Linear Transformation (DLT) calibration for achieving the merit value used to quantify calibration accuracy

Step 1: Solve the following equation: *coefficient * reference coordinates = sum*

Step 2: Least Square to create output calibration vector from coefficient and sum

Step 3: Calculate the merit value:  *merit values = calibration vector * coefficient - sum*

Step 4: Return merit as RMS of all individual point merits
