# Supplementary material for: Validation of machine vision and action sport cameras for 3D motion analysis model reconstruction
Source: Sci Rep. 2023 Nov 29;13:21015. doi: 10.1038/s41598-023-46937-9 (PMC10687061; doi:10.1038/s41598-023-46937-9)
Supplement: Supplementary file 2 — Supplementary Table S1. [file 41598_2023_46937_MOESM2_ESM.docx]

**TABLE S1**: Static and dynamic calibration mean and standard deviation (SD) merit scores of accuracy for the experimental camera systems

| **Camera system** | ***Merit Score Mean (SD) mm*** | |
| --- | --- | --- |
|  | ***Static calibration*** | ***Dynamic calibration*** |
| **V-4** | — | 0.15 (.08) |
| **ASC-1** | 1.49 (.22) | 1.61 (.32) |
| **ASC-2** | 1.31 (.51) | 1.74 (.38) |
| **ASC-3** | 1.56 (.25) | 1.76 (.39) |
| **MV-4** | 1.22 (.25) | 0.48 (.03) |

ASC-1 (3 GoPro 5s + 1 GoPro 4), ASC-2 (3 GoPro 5s + 1 GoPro 9), ASC-3 (3 GoPro 5s), MV-4 (4 Sentech USB 3.0).
