# Supplementary material for: Validation of machine vision and action sport cameras for 3D motion analysis model reconstruction
Source: Sci Rep. 2023 Nov 29;13:21015. doi: 10.1038/s41598-023-46937-9 (PMC10687061; doi:10.1038/s41598-023-46937-9)
Supplement: Supplementary file 3 — Supplementary Table S2. [file 41598_2023_46937_MOESM3_ESM.docx]

**TABLE S2**: Mean and standard deviation (SD) for the individual camera systems predicting lengths for Segment-1 and Segment-2.

| **Camera system** | ***Segment-1 Mean (SD) mm*** | | ***Segment-2 Mean (SD) mm*** | |
| --- | --- | --- | --- | --- |
|  | ***Static calibration*** | ***Dynamic calibration*** | ***Static calibration*** | ***Dynamic calibration*** |
| **V-8** | — | 145.37 (.02) | — | 287.86 (.08) |
| **V-4** | — | 145.41 (.02) | — | 288.59 (.03) |
| **ASC-1** | 147.33 (.05) | 144.63 (.04) | 293.19 (.06) | 286.72 (.08) |
| **ASC-2** | 146.81 (.13) | 144.34 (.05) | 292.34 (.11) | 286.12 (.20) |
| **ASC-3** | 147.36 (.09) | 144.64 (.08) | 293.11 (.08) | 285.26 (.14) |
| **MV-4** | 146.14 (.07) | 145.28 (.11) | 290.67 (.04) | 286.31 (.06) |

ASC-1 (3 GoPro 5s + 1 GoPro 4), ASC-2 (3 GoPro 5s + 1 GoPro 9), ASC-3 (3 GoPro 5s), MV-4 (4 Sentech USB 3.0).
